# An energy-aware and Q-learning-based area coverage for oil pipeline monitoring systems using sensors and Internet of Things

**DOI:** 10.1038/s41598-022-12181-w

**Published:** 2022-06-10

**Authors:** Amir Masoud Rahmani, Saqib Ali, Mazhar Hussain Malik, Efat Yousefpoor, Mohammad Sadegh Yousefpoor, Amir Mousavi, Faheem khan, Mehdi Hosseinzadeh

**Affiliations:** 1 Future Technology Research Center, National Yunlin University of Science and Technology, Yunlin, Taiwan; 2 Department of Information Systems, College of Economics and Political Science, Sultan Qaboos University, Al Khoudh, Muscat Oman; 3 HoD Computing and IT (CIT) Global College of Engineering and Technology, P.O Box 2546, CPO Ruwi 112, Muscat, Oman; 4 Department of Computer Engineering, Dezful Branch, Islamic Azad University, Dezful, Iran; 5 Faculty of Civil Engineering, Technische Universitat Dresden, 01069 Dresden, Germany; 6 John von Neumann Faculty of Informatics, Obuda University, Budapest, 1034 Hungary; 7 Artificial Intelligence Laboratory, Gachon University, Seongnam, Republic of Korea; 8 Mental Health Research Center, Psychosocial Health Research Institute, Iran University of Medical Sciences, Tehran, Iran; 9 Computer Science, University of Human Development, Sulaymaniyah, Iraq; 10 Institute of Information Engineering, Automation and Mathematics, Slovak University of Technology in Bratislava, Bratislava, Slovakia; 11 Institute of Information Society, University of Public Service, Budapest, 1083 Hungary

**Keywords:** Engineering, Mathematics and computing, Data mining, Data processing

## Abstract

Pipelines are the safest tools for transporting oil and gas. However, the environmental effects and sabotage of hostile people cause corrosion and decay of pipelines, which bring financial and environmental damages. Today, new technologies such as the Internet of Things (IoT) and wireless sensor networks (WSNs) can provide solutions to monitor and timely detect corrosion of oil pipelines. Coverage is a fundamental challenge in pipeline monitoring systems to timely detect and resolve oil leakage and pipeline corrosion. To ensure appropriate coverage on pipeline monitoring systems, one solution is to design a scheduling mechanism for nodes to reduce energy consumption. In this paper, we propose a reinforcement learning-based area coverage technique called CoWSN to intelligently monitor oil and gas pipelines. In CoWSN, the sensing range of each sensor node is converted to a digital matrix to estimate the overlap of this node with other neighboring nodes. Then, a Q-learning-based scheduling mechanism is designed to determine the activity time of sensor nodes based on their overlapping, energy, and distance to the base station. Finally, CoWSN can predict the death time of sensor nodes and replace them at the right time. This work does not allow to be disrupted the data transmission process between sensor nodes and BS. CoWSN is simulated using NS2. Then, our scheme is compared with three area coverage schemes, including the scheme of Rahmani et al., CCM-RL, and CCA according to several parameters, including the average number of active sensor nodes, coverage rate, energy consumption, and network lifetime. The simulation results show that CoWSN has a better performance than other methods.

## Introduction

**Figure 1:**
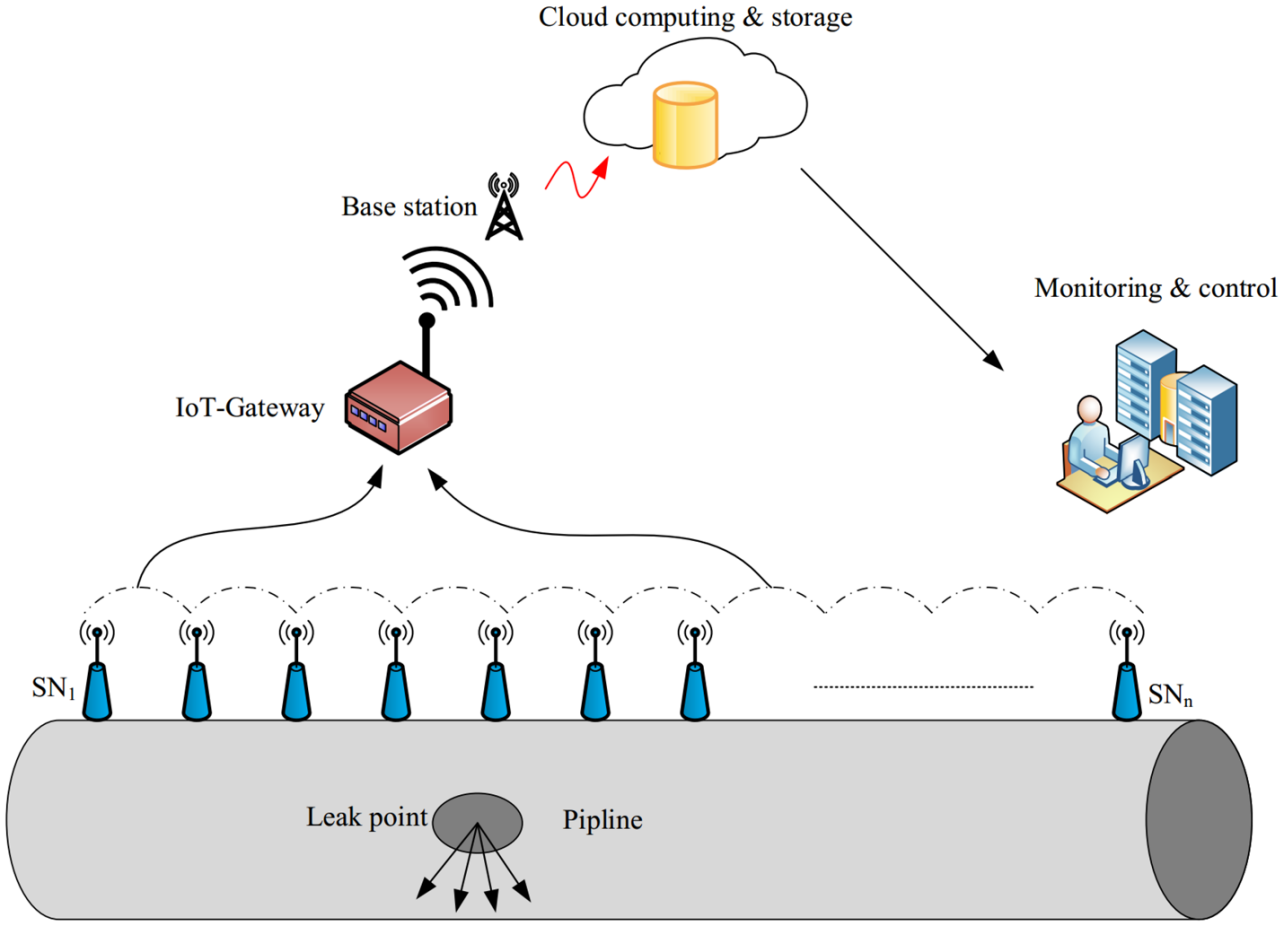
Smart oil pipeline.

Today, wireless sensor networks (WSNs) have become an attractive research subject for many researchers in industry and university^1,2^. WSNs consist of a high number of sensor nodes distributed in the network environment to monitor the Region of Interest (RoI)^3,4^. Initially, these networks were specifically designed for military applications^5,6^. However, the growing advances in these networks have led to new technologies such as the Internet of Things (IoT)^7,8^. The IoT has created new opportunities to communicate things around us, such as lamp switches^9^, oil and gas transmission pipelines, industrial machines^10^, home equipment^11^, cars, and the human body using the Internet platform^12,13^. Today, this new technology is applied in various industrial and engineering fields such as pipeline monitoring systems. Intelligent monitoring on oil pipelines is a combination of industry and IoT^14,15^. In recent years, smart pipeline monitoring systems are possible due to advances in low-consumption electrical circuits and small, low-consumption, and cheap electronic equipment production, such as smart sensors^16,17^. Figure 1 shows a smart oil pipeline. The purpose of smart pipelines is to collect different information about the health of the pipeline using heterogeneous sensors. Because pipeline systems are responsible for transporting oil and gas, any leakage in the pipeline can cause financial and environmental damage. Today, only some of the important points are controlled in a pipeline. These points are several kilometers away from each other. This monitoring method is ineffective and inflexible^17,18^. A smart pipeline can provide a better understanding of the pipeline network. In large-scale pipelines, different sensor nodes are installed on the pipeline so that they can collect and process various parameters such as temperature, pressure, humidity, audio, and contamination and send this information to the control center.

The sensor nodes sense the target or phenomena occurred in their sensing ranges and process the data collected from this area and send the information to BS directly or using a multi-hop manner^19–21^. When monitoring pipelines, the main challenge is energy consumption because sensor nodes are deployed in the soil. This creates restrictions such as poor data transmission and data loss^22,23^. Also, this underground environment imposes major limitations on sensor nodes, especially poor radio frequency (RF) so that the RF transmission range in soil is significantly lower than in air^24,25^. Therefore, communication between nodes is much more limited in the underground environment. Therefore, we must consider shorter communication ranges for sensor nodes. Furthermore, repair or replacement of nodes is very costly. As a result, sensor nodes should have a long lifetime and consume less energy because they have a small communication and sensing range and limited energy resources. Therefore, providing proper coverage and maintaining connections play a very important role in efficiency and optimal performance of smart oil pipelines.

Coverage means the region or point monitored by sensor nodes scattered in that environment. Sensor nodes cover a region or point when that point or region is inside their sensing ranges^26,27^. Therefore, if a large number of sensor nodes are installed on the pipeline, this pipeline is covered properly and reliably. However, the pipeline structure is very complicated and dynamic. As a result, it is very difficult to measure all parameters related to the pipeline structure such as pressure and temperature to detect the pipeline corrosion. Therefore, the full coverage of the pipeline structure is impossible. Thus, in each period, only a number of nodes installed on the pipeline are activated to partially monitor the pipeline and provide a proper coverage rate. Coverage schemes can be implemented in two forms, including centralized and decentralized (distributed). In centralized coverage methods, only BS manages the coverage operation in the network. While in distributed coverage schemes, sensor nodes collaboratively execute coverage operations. Also, coverage methods can be executed statically and dynamically. Static coverage schemes determine the locations of sensor nodes in the network deterministically before bootstrapping the network. Note that this strategy does not change over the network lifetime. Dynamic methods periodically update the status of sensor nodes in the network. This means that this coverage strategy changes throughout the network lifetime.

On the other hand, network lifetime is a very important issue when covering the network because this parameter specifies how long time the pipeline network can work properly to meet the coverage requirements. Therefore, the network lifetime is an important criterion for evaluating smart pipelines. A suitable solution for this issue is to design a scheduling mechanism for sensor nodes. In this mechanism, in each scheduling period, only part of sensor nodes are activated in the network, and other nodes are in sleep status to reduce their energy consumption. The active sensor nodes should guarantee the desired partial coverage rate. The scheduling issue in the network can be considered as an optimization issue. However, real-world optimization issues are very complicated because they are large and dynamic. As a result, it is necessary to use computational intelligence-based methods such as reinforcement learning (RL) to solve these issues^28,29^. Today, RL algorithms are being popular rapidly because they can successfully find optimal response at a proper time. RL is suitable for solving issues such as routing, data aggregation, and coverage in WSN and IoT. RL is an appropriate tool in computational intelligence. It can learn optimal policy through interaction with the environment^30,31^. In this paper, we use the RL algorithm to achieve proper coverage rate so that our scheme uses the minimum number of active sensor nodes and improves energy consumption in the network.

In this paper, we propose an appropriate area coverage method called CoWSN to intelligently monitor oil and gas pipelines, so that CoWSN balances energy consumption and increases coverage quality in the network. The main contributions of CoWSN are as follows:

- In CoWSN, sensing range of each sensor node is converted to the digital matrix using a new, efficient, and distributed technique. The digital matrix helps us to calculate the overlap of this node with other neighboring nodes using geometric mathematics.
- In CoWSN, a Q-Learning-based scheduling mechanism is presented to calculate the activity time of sensor nodes based on three parameters, including the overlap between a sensor node and neighboring nodes, energy, and distance to BS.
- In CoWSN, the replacement time of nodes is predicted using an appropriate technique so that the data transmission process between sensor nodes and the base station is not disrupted.

In the following, the paper is organized as follows: in “Related works”, the related works are expressed. Then, the basic concepts used in the proposed method are summarized in “Basic concepts”. “System model” describes the system model in CoWSN. “Proposed schemeProposed scheme” explains our proposed method in detail. “Simulation and result evaluation” compares the simulation results of CoWSN with other coverage schemes. Finally, the conclusion of the paper is presented in “Conclusion”.

## Related works

In^32^, the CCM-RL technique is presented in WSNs to maintain connections and coverage using reinforcement learning. CCM-RL tries to maximize coverage rate and maintain connections along with energy efficiency. In this method, nodes execute a learning algorithm to learn their optimal activity. As a result, CCM-RL activates a subset of nodes at a specific time. This reduces energy consumption in the network and provides an appropriate coverage rate and suitable connectivity. CCM-RL is a dynamic, distributed, and scalable coverage method. However, the scheduling mechanism has only taken attention to two parameters, including distance and coverage rate, and has ignored energy parameter. Also, CCM-RL has a lot of delay.

In^5^, an area coverage approach based on fuzzy logic (FL) and shuffled frog-leaping algorithm (SFLA) is proposed. This approach balances energy consumption, increases network lifetime and improves coverage quality. This method calculates the overlap between sensor nodes using a distributed digital matrix-based approach. Then, a fuzzy scheduling mechanism is designed. This mechanism considers three parameters, including overlap, residual energy, and distance between each node and the base station to determine the activity time of each sensor node. Moreover, this method uses a strategy to predict the death of sensor nodes and prevent holes in network. Finally, this approach uses SFLA to find the best replacement strategy, which covers the holes created in the network and maximizes coverage rate. This coverage method is distributed, dynamic, and scalable. However, this method has a high communication overhead due to fuzzy scheduling mechanism. Also, the use of SFLA increases delay in the network.

In^33^, the CCA technique is offered in two forms, including distributed and centralized, for homogeneous WSNs. CCA solves the *k*-coverage issue when deploying sensor nodes in the network. For solving this issue, CCA tries to use the lowest number of sensor nodes and increase network lifetime. CCA designs a scheduling process. According to this process, a subset of nodes is selected for covering the desired area. Centralized CCA can be implemented dynamically and statically. The dynamic method has a greater time complexity in comparison with the static method. Also, the dynamic approach has a better coverage rate than the static scheme. In general, distributed CCA is more scalable than centralized CCA because the distributed scheme relies on local information. Of course, distributed CCA has a lot of communication overhead.

In^34^, a partial coverage technique based on learning automata (PCLA) is suggested in WSNs. PCLA solves the coverage issue and uses the minimum number of sensor nodes and maintains appropriate connectivity. This method uses learning automata (LA) to determine the activity time of nodes in the network. PCLA creates a backbone in the network. Therefore, a subset of the sensor nodes is selected for covering the RoI and maintaining connectivity. PCLA is a distributed, dynamic, and scalable method. However, this method has a lot of overhead.

In^35^, a coverage method based on the genetic algorithm called MIGA is presented in heterogeneous WSNs. This scheme is inspired by IGA. MIGA presents a function to estimate the area coverage. However, this function is incomplete and does not consider the various overlap scenarios when overlapping two nodes. MIGA includes five components: individual representation, population initialization, genetic operators, fitness function, and VFA optimization. MIGA achieves a high-quality response for maximizing coverage rate. However, MIGA is a centralized coverage method. It has a lot of computational overhead and is not scalable.

In^36^, the maximum coverage sets scheduling (MCSS) mechanism is presented in WSN. MCSS schedules the coverage sets and improves network lifetime. This method uses a greedy algorithm for searching the problem. MCSS has acceptable time complexity and computational complexity. MCSS assumes that coverage sets and time slots of nodes are predetermined. MCSS is a centralized method and is not scalable. It takes into account only the activity time of nodes and does not consider other parameters such as energy and distance.

In^37^, a barrier coverage method is offered in homogeneous wireless sensor networks. It uses the minimum number of sensor nodes when covering the network. In this method, the authors calculate the overlap between sensor nodes based on the angle between their sensing ranges. Furthermore, this method detects failed nodes and covers the holes created in the network. The most important advantage of this method is the proper coverage rate with the lowest sensor nodes. However, the authors have only taken attention to two parameters, including distance and overlap. Also, this method cannot predict the death time of nodes in the network. This scheme increases the latency in the network.

In^38^, two coverage schemes are proposed for heterogeneous wireless sensor networks. These schemes use the improved cuckoo search (ICS) algorithm and chaotic flower pollination algorithm (CFPA). These methods try to reduce the implementation cost and energy consumption in the network. This method presents a fitness function, which considers only one parameter, including the overlap between nodes. It can be improved by considering more parameters. ICS and CFPA have a small computational complexity. They are simple and fast (high convergence speed) and can achieve a high-quality response. Furthermore, these schemes are static. This means that they explore the best replacement for sensor nodes and this strategy is fixed over network life.

In^39^, two area coverage schemes are suggested in WSN. These approaches utilize the genetic algorithm (GA) and particle swarm optimization (PSO). The authors assume that the network consists of a number of obstacles. Then, they define the coverage issue in this network and use GA and PSO for addressing it. The important advantage of these approaches is to obtain the highest coverage rate with suitable computational overhead. However, this method considers only one parameter (i.e. the overlap between sensor nodes) when designing the cost function and does not consider other parameters such as energy. Also, this method focuses only on maximum area coverage and does not consider network lifetime. It is centralized and static. This reduces scalability.

In^40^, a mathematical model is proposed to solve the coverage issue in WSN. This method moves sensor nodes toward low-density network areas to maximize the coverage rate in the network. This method distributes the sensor nodes in the network evenly. It uses an improved version of the virtual force algorithm. This method has low computational complexity. However, it is static and centralized and is not scalable. In this method, the goal is to maximize area coverage and does not pay attention to network lifetime.

## Basic concepts

In this section, we briefly describe a well-known reinforcement learning technique called Q-Learning because we use this technique in the proposed method for designing the scheduling mechanism.

### Q-Learning

Reinforcement learning (RL) allows machines or agents to learn their ideal behavior in a particular situation based on previous experience^28^. A RL-based model learns through interaction with the environment and collects information to do a specific activity. Q-Learning is a model-free and off-policy reinforcement algorithm. Q-Learning helps one agent to learn its optimal actions. According to this learning algorithm, state-action pairs are stored in a table called Q-table. This table receives a state-action pair as input and returns the Q-value as output. In Q-Learning, the goal is to maximize the Q-value. To achieve this goal, the agent adjusts its action strategy according to the reward received from the environment’s feedback. In the learning process, the agent evaluates how many an action is suitable in the current state to choose a better action in the next iteration. Q-value is updated in each iteration using Eq. (1):

1 $$\begin{aligned} Q\left( {s_{t}},{a_{t}} \right) \leftarrow Q\left( {s_{t}},{a_{t}} \right) +\alpha \left[ {r_{t+1}}+\gamma \underset{a}{\mathop {\max }}\,Q\left( {s_{t+1}},a\right) -Q\left( {s_{t}},{a_{t}} \right) \right] \end{aligned}$$

where *t* is current iteration, $a\in A$ is the action set, ${r_{t+1}}$ indicates the reward value received by the agent after doing the action ${a_{t}}$ in the state ${s_{t}}$. When the agent performs the action ${a_{t}}$, its state changes from ${s_{t}}$ to ${s_{t+1}}$. $\underset{a}{\mathop {\max }}\,Q\left( {s_{t+1}},a\right)$ indicates maximum Q value when the agent performs the action *a* in the next iteration. $0<\alpha \le 1$ is the learning rate. If $\alpha =0$, then the agent does not learn anything. If $\alpha =1$, the agent learns only the last experience. In the proposed method, we consider $\alpha =0.1$. Also, $0<\gamma \le 1$ indicates the discount factor. It represents the reward importance. In fact, it indicates the agent’s effort for discovering the environment. We consider $\gamma =0.7$ in the proposed method. Note that there are two techniques, including $\epsilon$-greedy and Boltzmann in reinforcement learning to create a balance between exploration and exploitation^28^. In $\epsilon$-greedy, the quantitative allocation strategy with a small value $\epsilon$ is used for exploring. In the proposed method, we used the $\epsilon$-greedy technique to create a balance between exploration and exploitation.

## System model

This section consists of four subsections: network model, energy model, sensing model, and communication model. In the following, we describe each subsection in detail.

### Network model

In the proposed method, we consider a heterogeneous network with *N* sensor nodes. The nodes are heterogeneous. This means that they have different energy resources, sensing ranges, and communication ranges. They are randomly distributed in the network environment. Sensor nodes are equipped with a positioning system. Therefore, they are aware of their spatial coordinates $\left( {x_{i}},{y_{i}}\right)$ in the network. Also, the location of the base station $\left( {x_{BS}},{y_{BS}}\right)$ is known for each node in the network. Each sensor node knows its remaining energy (${E_{residual}}$) at any moment. If sensor nodes are in communication ranges of each other, they can directly communicate with each other through a wireless communication channel. In this model, the network includes one base station, ${N_{Static}}$ static sensor nodes, and ${N_{Dynamic}}$ mobile sensor nodes. Where,

2 $$\begin{aligned} {N_{Static}}+{N_{Dynamic}}=N \end{aligned}$$

And,

3 $$\begin{aligned} {N_{Dynamic}}\ll {N_{Static}} \end{aligned}$$

In the following, we describe the task of each node:

- Base station (BS): This node is responsible for receiving and processing information of sensor nodes.
- Static sensor nodes: These nodes are responsible for sensing the RoI and sending the sensed data to the base station.
- Mobile sensor nodes: These nodes are responsible for covering holes caused by the death of sensor nodes in the network.

### Energy model

In CoWSN, when a transmitter node such as $S{N_{i}}$ sends its data (*k* bits) to a receiver node like $S{N_{j}}$ and the distance between the two nodes is equal to *d*. $S{N_{i}}$ calculates the energy consumed for sending *k* bits according to Eq. (4):

4 $$\begin{aligned} {E_{TX}}\left( k,d\right) =\left\{ \begin{matrix} {E_{elec}}\times k+{{E}_{fs}}\times k+{{d}^{2}},\,\,\,\,\,\,\,d<{{d}_{0}} \\ {E_{elec}}\times k+{{E}_{mp}}\times k+{{d}^{4}},\,\,\,\,\,\,\,d\ge {{d}_{0}} \\ \end{matrix} \right. \end{aligned}$$

Also, $S{N_{j}}$ calculates the energy consumed for receiving *k* bits using Eq. (5):

5 $$\begin{aligned} {E_{RX}}\left( k,d \right) ={E_{elec}}\times k \end{aligned}$$

where ${E_{elec}}$ indicates the energy used by transmitter/receiver circuit, ${E_{fs}}$ and ${E_{mp}}$ are the energy required for the transmitter amplifier in the free space and multipath models, respectively. Equation (6) computes ${d_{0}}$, which is the threshold of transmission distance:

6 $$\begin{aligned} {d_{0}}=\sqrt{\frac{{E_{fs}}}{{E_{mp}}}} \end{aligned}$$

### Sensing model

CoWSN uses the binary sensing model that is also called 0/1 model. In this model, each $SN_{i}$ with spatial coordinates $\left( {x_{i}},{y_{i}}\right)$ can sense the circular area. The radius of this area is equal to $RS_{i}$. Consider one point in the RoI, for example $P=\left( {x_{p}},{y_{p}}\right)$. In binary sensing model, $SN_{i}$ can sense *P* only when their Euclidean distance is lower than radius $RS_{i}$. In this case, we state that this point is inside the sensing range of $SN_{i}$. Otherwise, *P* is outside the sensing range of $SN_{i}$ and cannot be covered by this node. This issue is expressed in Eq. (7):

7 $$\begin{aligned} C\left( S,P \right) =\left\{ \begin{matrix} 1\,\,\,\,\,d\left( SN_{i},P \right) \le RS_{i} \\ 0\,\,\,\,\,d\left( SN_{i},P \right) >RS_{i} \\ \end{matrix} \right. \end{aligned}$$

And, the distance between $SN_{i}$ and *P* is shown by $d\left( SN_{i},P\right)$. This parameter is calculated by Eq. (8):

8 $$\begin{aligned} d\left( SN_{i},P \right) =\sqrt{{{\left( {x_{i}}-{x_{p}}\right) }^{2}}+{{\left( {y_{i}}-{y_{p}} \right) }^{2}}} \end{aligned}$$

### Communication model

CoWSN uses the binary disk model as the communication model. This model is similar to the sensing model. According to the communication model, the communication radius $\left( RC\right)$ is known as the upper communication bound. This means that if there are two nodes that are in the communication ranges of each other, they can communicate directly together. Note that communication radius $\left( RC\right)$ is greater than sensing radius $\left( RS\right)$, so that:

9 $$\begin{aligned} RS<RC \end{aligned}$$

## Proposed scheme

Our scheme (CoWSN) is an area coverage technique. It increases the coverage quality, balances energy consumption in the network, and improves network lifetime. CoWSN consists of three parts:

- Converting sensing ranges of sensor nodes to digital matrix
- Q-learning-based scheduling mechanism
- Node replacement

### Converting sensing ranges of sensor nodes to digital matrix

In this section, a decentralized technique is introduced for calculating the overlap of sensor nodes with their neighbors. According to this technique, the sensing range of a node is converted into a digital matrix. Note that a matrix is known as a digital matrix if all elements are zero or one. In the following, we describe this process in detail.

First, the sensing range of a node (for example, $SN_{i}$) is converted into a digital matrix. In this process, the node’s coordinates is considered as the pole in the polar coordinate system and the sensing range of this node is displayed as a circular area $C_{i}$ with the sensing radius $RS_{i}$. $C_{i}$ is divided into *n* sectors (${\text {s}}\overset{\scriptscriptstyle \frown }{e}{{c}_{p}},\,\,\,p=1,2,\ldots ,n$) and *m* smaller circles (${c_{q}},\,\,\,q=1,2,\ldots ,m$) with the same center. So that, $n=\frac{2\pi }{\Delta \theta }$ and $m=\frac{RS_{i}}{\Delta R}$. Also, $\Delta \theta$ is the angle of each ${\text {s}}{\hat{e}}{c_{p}}$. Furthermore, each circle ${c_{q}}$ has a radius such as ${r_{q}}$, this process is shown in Eqs. (10) and (11):

10 $$\begin{aligned} C_{i}= & {} \left\{ \begin{matrix} {\text {s}}\overset{\scriptscriptstyle \frown }{e}{c_{1}}:\theta \le s{\hat{e}}{c_{1}}\le \theta +\Delta \theta \\ {\text {s}}\overset{\scriptscriptstyle \frown }{e}{c_{2}}:\theta +\Delta \theta \le s{\hat{e}}{c_{2}}\le \theta +2\Delta \theta \\ \vdots \\ {\text {s}}\overset{\scriptscriptstyle \frown }{e}{c_{n}}:\theta +\left( n-1\right) \Delta \theta \le s{\hat{e}}{c_{n}}\le \theta +n\Delta \theta \\ \end{matrix}\right. \end{aligned}$$

11 $$\begin{aligned} C_{i}= & {} \left\{ \begin{matrix} c_{1}:\,{r_{1}}=m\Delta R \\ c_{2}:{r_{2}}=\left( m-1 \right) \Delta R \\ \vdots \\ c_{m}:{r_{m}}=\Delta R \\ \end{matrix} \right. \end{aligned}$$

**Figure 2:**
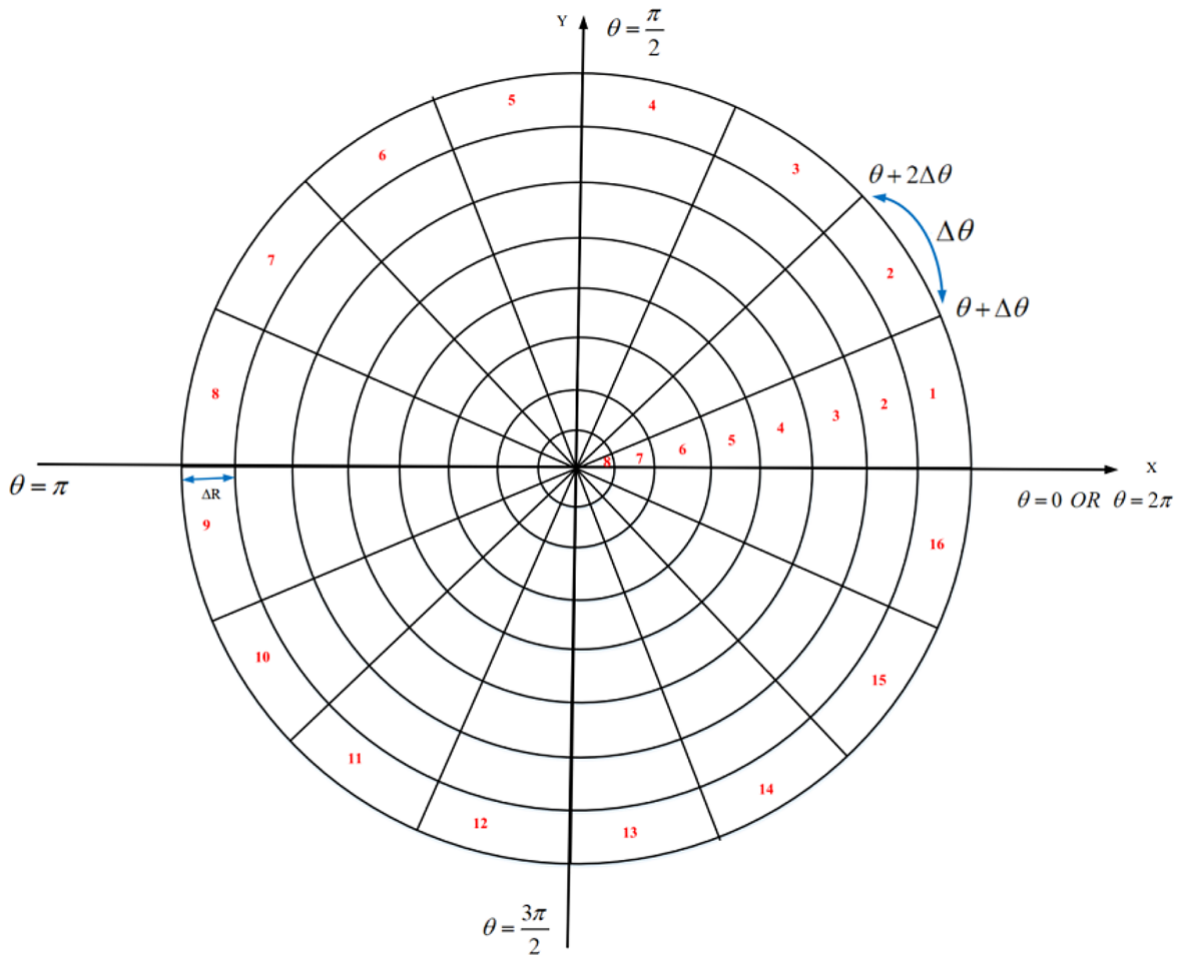
Dividing the sensing range of a sensor node into small rectangular sections.

Figure 2 shows an example in which ${C_{i}}$ is divided into 16 sectors and 8 smaller circles. According to Fig. 2, this process partitions ${C_{i}}$ into small rectangular sections. Note that $\Delta \theta$ and $\Delta R$ can be adjusted based on the problem requirements. When the user selects $\Delta \theta$ and $\Delta R$ close to zero, the result will be more accurate. However, this work increases memory consumption.

**Figure 3:**
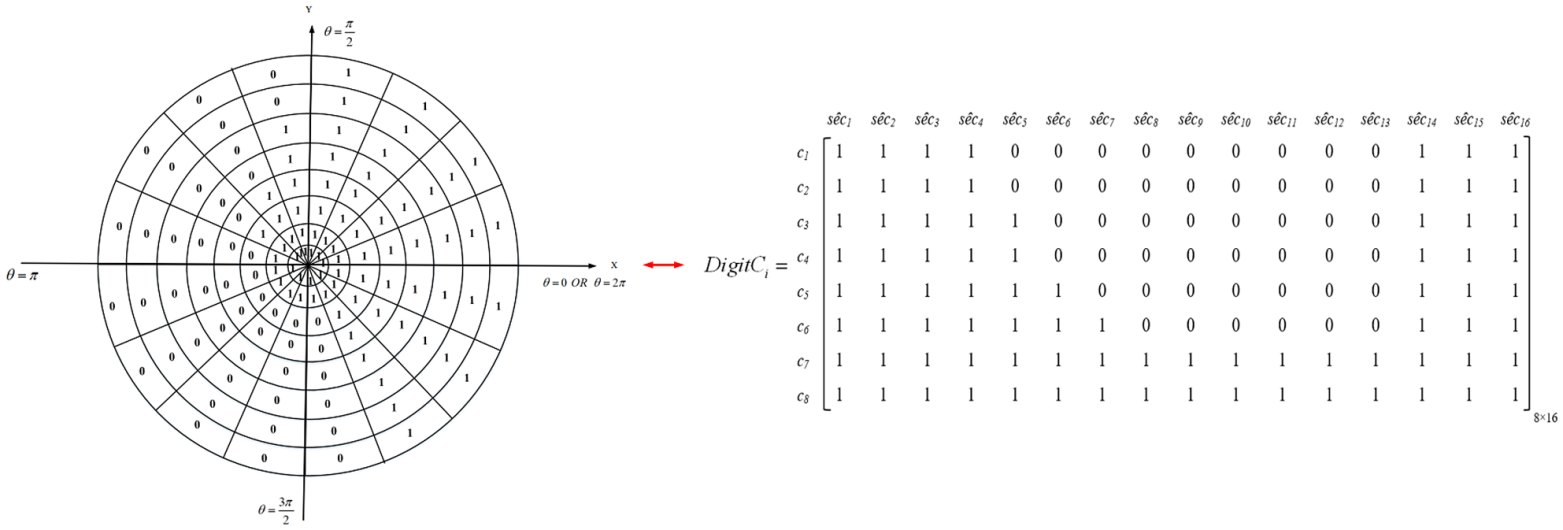
Digital matrix corresponding to the sensing range of the sensor node.

Now, ${C_{i}}$ is converted to an $m\times n$ digital matrix. In this matrix, rows and columns are equal to smaller circles ($c_{q}$) and sectors (${\text {s}}\overset{\scriptscriptstyle \frown }{e}{{c}_{p}},\,\,\,p=1,2,\ldots ,n$), respectively. Furthermore, matrix elements ($a_{qp}$) represent rectangular sections, so that $q=1,\ldots ,m$ and $p=1,\ldots ,n$. Note that $a_{qp}$ can be one or zero. In fact, $a_{qp}$ is equal to one when the corresponding rectangular section overlaps with the sensing range at least one neighboring node. Otherwise, $a_{qp}$ is equal to zero. Note that $a_{qp}$ will be zero, when the corresponding rectangular section is not fully covered by neighboring nodes. An example of this digital matrix is shown in Fig. 3.

In the following, we describe how to determine the value of $a_{qp}$. In the first step, each sensor node like $SN_{i}$ shares its information, including its identifier ($ID_{i}$), spatial coordinates $\left( {x_{i}},{y_{i}}\right)$, remaining energy ($E_{residual_{i}}$) and sensing radius $\left( RS_{i}\right)$, with its own neighbors and broadcasts a Hello message for them. Then, the node stores the information of its neighbors in a table called $Table_{neighbor}$, which is shown in Table 1.

**Table 1:** $Table_{neighbor}$ stored in $SN_{i}$.

Number	ID	Spatial coordinates	Sensing radius	Remaining energy	Scheduling state
1	$ID_{j}$	$\left( {x_{j}},{y_{j}}\right)$	$RS_{j}$	${E_{residual_{j}}}$	${T_{Scheduling_{j}}}$
2	$ID_{k}$	$\left( {x_{k}},{y_{k}}\right)$	$RS_{k}$	${E_{residual_{k}}}$	${T_{Scheduling_{k}}}$

Now, $SN_{i}$ can obtain the Euclidean distance between itself and its neighbors such as $SN_{j}$ according to Eq. (12):

12 $$\begin{aligned} {d_{ij}}=\sqrt{{{\left( {x_{i}}-{x_{j}}\right) }^{2}}+{{\left( {y_{i}}-{y_{j}}\right) }^{2}}} \end{aligned}$$

So that $\left( {x_{i}},{y_{i}}\right)$ and $\left( {{x}_{j}},{y_{j}}\right)$ are coordinates $SN_{i}$ and $SN_{j}$, respectively.

If ${d_{ij}}\le RS_{j}-RS_{i}$ or $RS_{i}-RS_{j}<d_{ij}<RS_{i}+RS_{j}$, then two nodes overlap in their sensing range. This means that if $d_{ij}\le RS_{j}-RS_{i}$ then all $a_{qp}$ where, $q=1,\ldots ,m$ and $p=1,\ldots ,n$, will be one. Otherwise, if $RS_{i}-RS_{j}<d_{ij}<RS_{i}+RS_{j}$, then the angle of $C_{j}$ with regard to the pole (i.e. $SN_{i}$) in the polar coordinate system is calculated using Eq. (13):

13 $$\begin{aligned} \alpha =\arctan \left( \frac{{x_{j}}-{x_{i}}}{{y_{j}}-{y_{i}}}\right) ,\,\,\,\,\,\,0\le \alpha \le 2\pi \end{aligned}$$

See Fig. 4.

**Figure 4:**
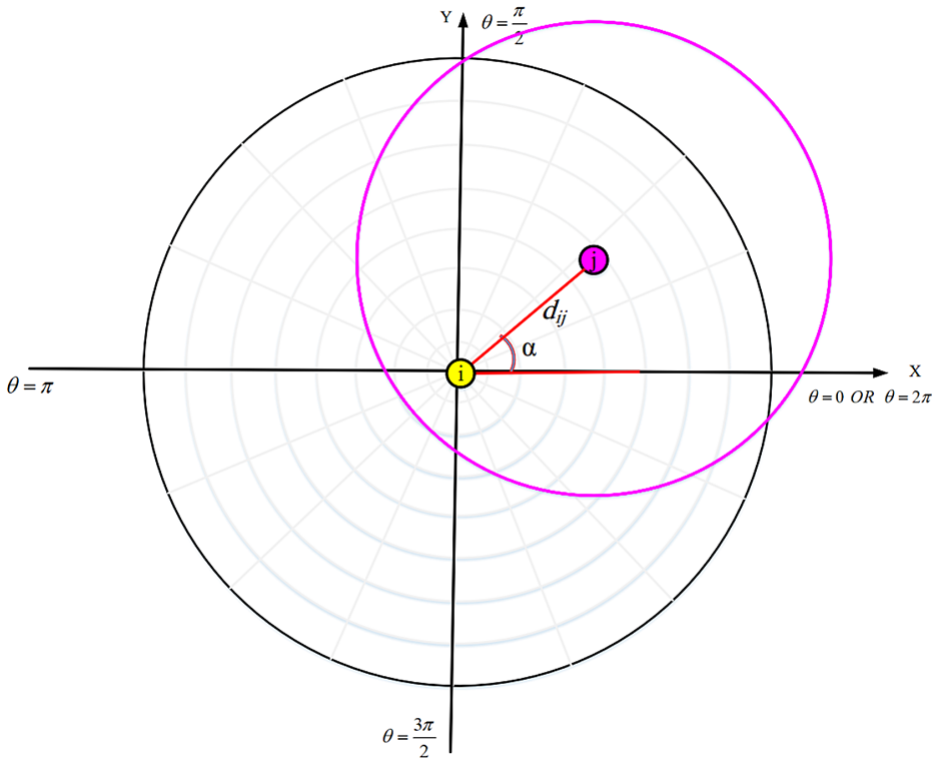
The angle of $SN_{j}$ with regard to $SN_{i}$.

For obtaining the value of $a_{qp}$ in the digital matrix and calculating the overlap between $SN_{i}$ and $SN_{j}$, we follow the following commands.

**Figure 5:**
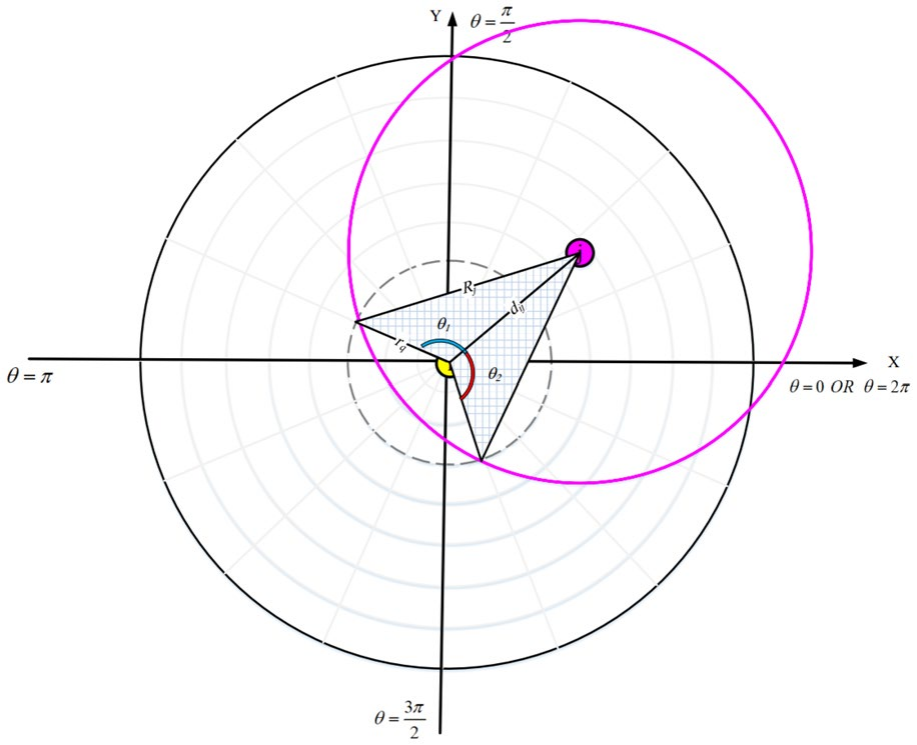
Overlapping area between $c_{q}$ and $C_{j}$.

- If ${d_{ij}}\ge {R_{j}}+{r_{q}}$, so that $1\le q\le 8$ in this example, $c_{q}$ and all smaller circles are outside $C_{j}$. As a result, $a_{qp}$ will be zero in the corresponding rows.
- If ${d_{ij}}\le {R_{j}}-{r_{q}}$, so that $1\le q\le 8$ in this example, $c_{q}$ and all smaller circles are inside $C_{j}$. Therefore, $a_{qp}$ will be one in the corresponding rows.
- If ${R_{j}}-{r_{q}}<{d_{ij}}<{R_{j}}+{r_{q}}$, $c_{q}$ and $C_{j}$ partially overlap with each other, and this overlap is calculated as follows:
  - As shown in Fig. 5, a triangle with three vertices, $\left( {x_{i}},{y_{i}}\right)$, $\left( {x_{j}},{y_{j}}\right)$, and the intersection point of $c_{q}$ and $C_{j}$ is considered. Now, compute the length of three sides of this triangle.
  - Here, the angle ${{\theta }_{1}}={{\theta }_{2}}$ is obtained using the cosine law:
    14 $$\begin{aligned} R_{j}^{2}=r_{q}^{2}+d_{ij}^{2}-2{r_{q}}{d_{ij}}\cos {{\theta }_{1}} \end{aligned}$$
    where,
    15 $$\begin{aligned} {{\theta }_{1}}=\arccos \left( \frac{r_{q}^{2}+d_{ij}^{2}-R_{j}^{2}}{2{r_{q}}{d_{ij}}}\right) ,\,\,\,\,\,\,0\le {{\theta }_{1}}\le \pi \end{aligned}$$
  - Equation (16) computes the overlapping area (${{\gamma }_{q}}$) between $c_{q}$ and $C_{j}$:
    16 $$\begin{aligned} \alpha -{{\theta }_{1}}\le {{\gamma }_{q}}\le \alpha +{{\theta }_{1}},\,\,\,\,\,\,\,\,\,\,0\le {{\gamma }_{k}}\le 2\pi \end{aligned}$$
  - Now, Eq. (17) computes $a_{qp}$ corresponding to the row $c_{q}$ and the sector ${{\sec }_{p}}$.
    17 $$\begin{aligned} {a_{qp}}=\left\{ \begin{matrix} 1\,\,,\,\,\,IF\,\,{{R}_{j}}-{{r}_{q}}<{{d}_{ij}}<{{R}_{j}}+{{r}_{q}}\,\,AND\,\,\,\alpha -{{\theta }_{1}}\le {\text {s}}{\hat{e}}{{c}_{p}}\le \alpha +{{\theta }_{1}} \\ 0\,\,,\,\,\,\,\,\,\,\,\,\,\,\,\,\,\,\,\,\,\,\,\,\,\,\,otherwise\,\,\,\,\,\,\,\,\,\,\,\,\,\,\,\,\,\,\,\,\,\,\,\,\,\,\,\,\,\,\,\,\,\,\,\,\,\,\,\,\,\,\,\,\,\,\,\,\,\,\,\,\,\,\,\,\,\,\,\,\,\,\,\,\,\,\,\,\,\,\,\,\,\,\, \\ \end{matrix}\right. \end{aligned}$$

This process is repeated for all $c_{q}$, $1\le q\le 8$ to calculate all $a_{qp}$, where, $q=1,\ldots ,m$ and $p=1,\ldots ,n$. Algorithm 1 presents the pseudocode of this process.
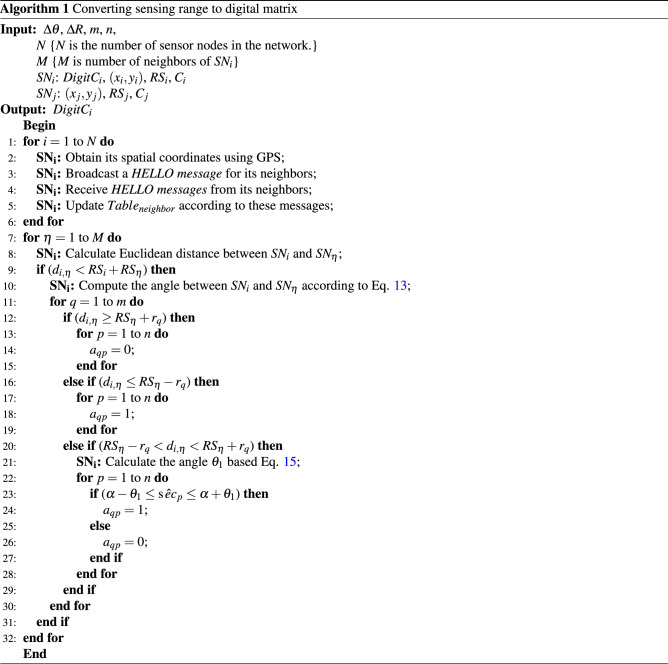


### Q-learning-based scheduling mechanism

In this section, our goal is to design a Q-learning-based scheduling mechanism so that each sensor node learns independently and automatically the best ON/OFF time slots in any scheduling round ($T_{Scheduling}$) to maximize coverage rate and network lifetime. The learning process immediately begins after deploying sensor nodes in the network. At the start time ($t=0$), we initialize the Q-learning parameters, including learning rates ($\alpha$), discount factor ($\gamma$), and Q value. Also, all sensor nodes are activated at $t=0$. Then, the time slots are updated and modified in the learning process according to the Q-learning algorithm to achieve optimal response. As stated in “Converting sensing ranges of sensor nodes to digital matrix”, at $t=0$, each sensor node shares its information such as its identifier ($ID_{i}$), spatial coordinates $\left( {x_{i}},{y_{i}}\right)$, remaining energy ($E_{residual_{i}}$), the scheduling state ($T_{Scheduling}$), and sensing radius $RS_{i}$ with its own neighbors, and stores their information in $Table_{neighbor}$ shown in Table 2. In the learning process, this information is used.

In the following, we describe various components in this scheduling mechanism:

**Agent** In this protocol, each sensor node ($SN_{i}$) plays the agent role.

**Environment** In this learning issue, the network plays the role of environment.

**Figure 6:**
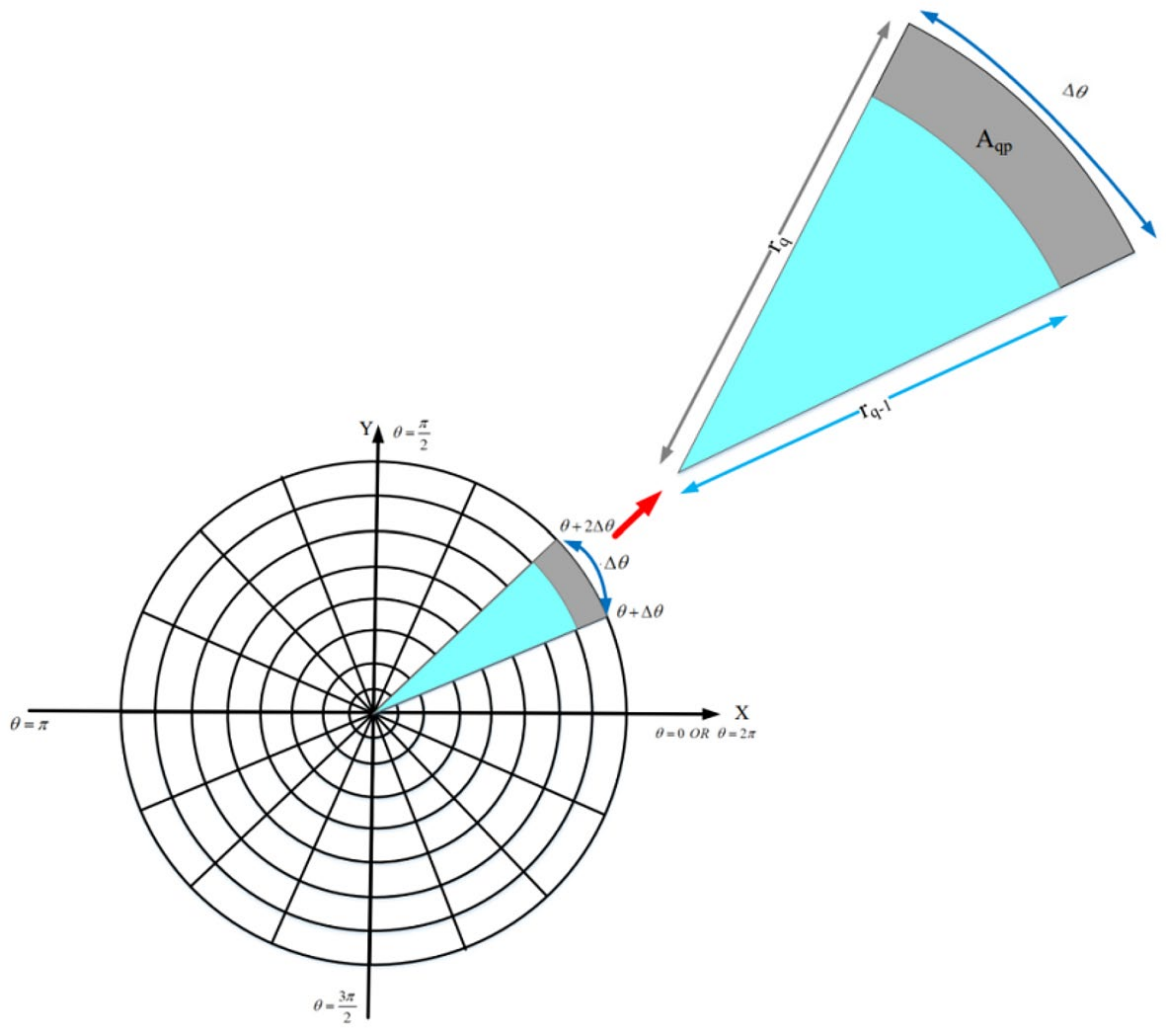
The calculation of $A_{qp}$ area.

**State** In this issue, the state of an agent is the overlap value of this agent with other active neighbors. The overlapping area ($O_{i}$) of each node is calculated using the digital matrix. First, the rectangular area $A_{qp}$ (Gray area shown in Fig. 6) is calculated based on Eq. (18):

18 $$\begin{aligned} {A_{qp}}=Area_{{Circle\,sector}_{_{qp}}}-Area_{{Circle\,sector}_{_{\left( q-1 \right) p}}} \end{aligned}$$

where $Area_{{Circle\,sector}_{_{qp}}}$ is the sector area *p* in the circle $c_{q}$. It is calculated through Eq. (19):

19 $$\begin{aligned} Area_{{Circle\,sector}_{_{qp}}}=\frac{1}{2}r_{q}^{2}\Delta \theta \end{aligned}$$

After merging Eqs. (19) and (18), we have:

20 $$\begin{aligned} {A_{qp}}=\frac{1}{2}r_{q}^{2}\Delta \theta -\frac{1}{2}r_{q-1}^{2}\Delta \theta =\frac{1}{2}\Delta \theta \left( r_{q}^{2}-r_{q-1}^{2}\right) \end{aligned}$$

As stated in Eq. (11):

21 $$\begin{aligned} \begin{array}{l} {r_{q}}=\left( m-\left( q-1 \right) \right) \Delta R \\ {r_{q-1}}=\left( m-\left( q-2 \right) \right) \Delta R \\ \end{array} \end{aligned}$$

As a result, $A_{qp}$ is equal to Eq. (22):

22 $$\begin{aligned} {{A}_{qp}}= & {} \frac{1}{2}\Delta \theta \,\Delta {{R}^{2}}\left( {{\left( m-\left( q-1 \right) \right) }^{2}}-{{\left( m-\left( q-2 \right) \right) }^{2}} \right) \nonumber \\= & {} \frac{1}{2}\Delta \theta \,\Delta {{R}^{2}}\left( 2\left( q-m \right) -3 \right) \end{aligned}$$

Therefore, $O_{i}$ is obtained according to Eq. (23):

23 $$\begin{aligned} {O_{i}}=\frac{\sum \nolimits _{q=1}^{m}{\sum \nolimits _{p=1}^{n}{\frac{1}{2}\left( {{a}_{qp}} \right) \Delta \theta \,\Delta {{R}^{2}}\left( 2\left( q-m \right) -3\right) }}}{\pi RS_{i}^{2}} \end{aligned}$$

where $m\times n$ is the size of $DigitC_{i}$ and $RS_{i}$ represents the sensing radius of $SN_{i}$.

**Action** In the scheduling issue, the action is a set of all activities that can be done by the agent $SN_{i}$ in the state $O_{i}$. Assume that the scheduling round ($T_{Scheduling}$) includes ten time slots ($Ts_{i},\,\,\,\,\,\,i=1,\ldots ,10$). Each sensor node can be active in some of these time slots (i.e. $Ts_{i}=T_{ON}$) or it can be sleep in other time slots ($Ts_{i}=T_{OFF}$) so that $T_{Scheduling}=\sum \nolimits _{i=1}^{10}{Ts_{i}}$. To better understand this issue, consider the example presented in Table 2. The purpose of the learning algorithm is to find the best possible scheduling for each sensor node $SN_{i}$. As a result, the action corresponding to $SN_{i}$ is as $T_{Scheduling}^{t}={{\left[ Ts_{1},Ts_{2},\ldots ,Ts_{10}\right] }^{t}}$ at the iteration *t*.

**Table 2:** Time slots corresponding to $SN_{i}$.

$$T_{Scheduling}$$									
$Ts_{1}$	$Ts_{1}$	$Ts_{1}$	$Ts_{1}$	$Ts_{1}$	$Ts_{1}$	$Ts_{1}$	$Ts_{1}$	$Ts_{1}$	$Ts_{1}$
$T_{OFF}$	$T_{OFF}$	$T_{ON}$	$T_{ON}$	$T_{ON}$	$T_{OFF}$	$T_{OFF}$	$T_{ON}$	$T_{ON}$	$T_{ON}$

**Award** The award indicates the environment’s feedback with regard to the action performed by the agent $SN_{i}$ in the state $O_{i}$. If this action ($T_{Scheduling}$) is successful, the environment has positive feedback. Otherwise, it has negative feedback. In CoWSN, we consider two parameters for calculating the award function: the remaining energy ($E_{residual}$) and the distance between each node and the BS ($D_{i-BS}$). The reason for choosing these parameters is that high-energy nodes receive a positive award from the environment and increase their Q-value to stay in the ON mode for more time slots. Also, low-energy nodes receive a negative reward and decrease their Q-value to do their activities in fewer time slots. As a result, we choose the energy parameter to balance energy consumption in the network. Also, the purpose of choosing the distance parameter is that the sensor nodes close to the BS receive a positive reward from the environment and increase their Q-value to stay in the ON mode at more time slots because these nodes do more operations than other nodes. Therefore, the award function is calculated using Eq. (24):

24 $$\begin{aligned} r\left( O_{_{i}}^{t},T_{Scheduling}^{t} \right) =\left\{ \begin{array}{l} {{r}_{\min }},\,\,\,\,\,\,\,\,\,\,\,\,\,\,\,\,\,\,\,\,\,\,\,\,\,\,\,\,\,\,\,\,\frac{4}{5}\le O_{i}^{t+1}\le 1 \\ \left( \omega \left( 1-\frac{\sqrt{{{\left( {{x}_{i}}-{{x}_{BS}} \right) }^{2}}+{{\left( {{y}_{i}}-{{y}_{BS}}\right) }^{2}}}-{{\mu }_{d}}}{{{\sigma }_{d}}} \right) +\left( 1-\omega \right) \left( \frac{{{E}_{residual}}-{{\mu }_{e}}}{{{\sigma }_{e}}}\right) \right) ,\frac{2}{5}<O_{i}^{t+1}<\frac{4}{5} \\ {{r}_{\max }},\,\,\,\,\,\,\,\,\,\,\,\,\,\,\,\,\,\,\,\,\,\,\,\,\,\,\,\,\,\,\,0\le O_{i}^{t+1}\le \frac{2}{5} \\ \end{array}\right. \end{aligned}$$

In Eq. (24), $\left( {x_{i}},{y_{i}}\right)$ and $\left( {x_{BS}},{y_{BS}}\right)$ are the spatial coordinates of $SN_{i}$ and BS, respectively. ${{\mu }_{d}}$ indicates the average distance of active neighboring nodes to the BS obtained from $Table_{neighbor}$ of $SN_{i}$. Furthermore, ${{\sigma }_{d}}$ is the distance standard deviation of active neighbors at the current iteration. Also, $E_{residual}$ indicates the remaining energy of $SN_{i}$. ${{\mu }_{e}}$ and ${{\sigma }_{e}}$ are the average energy of active neighbors and the energy standard deviation of active neighbors at the current iteration, respectively. $\omega$ is a weight coefficient.

25 $$\begin{aligned} {{\mu }_{d}}= & {} \frac{1}{m}\sum \limits _{j=1}^{m}{\sqrt{{{\left( {x_{j}}-{x_{BS}}\right) }^{2}}+{{\left( {y_{j}}-{y_{BS}}\right) }^{2}}}} \end{aligned}$$

26 $$\begin{aligned} {{\sigma }_{d}}= & {} \sqrt{\frac{1}{m}\sum \limits _{j=1}^{m}{{{\left( \sqrt{{{\left( {x_{j}}-{x_{BS}} \right) }^{2}}+{{\left( {y_{j}}-{y_{BS}}\right) }^{2}}}-{{\mu }_{d}}\right) }^{2}}}} \end{aligned}$$

27 $$\begin{aligned} {{\mu }_{e}}= & {} \frac{1}{m}\sum \limits _{j=1}^{m}{{E_{residual_{j}}}} \end{aligned}$$

28 $$\begin{aligned} {{\sigma }_{e}}= & {} \sqrt{\frac{1}{m}\sum \limits _{j=1}^{m}{{{\left( {E_{residual_{j}}}-{{\mu }_{e}} \right) }^{2}}}} \end{aligned}$$

In Eqs. (25), (26), (27) and (28), *m* is the number of active neighboring nodes of $SN_{i}$ at the current iteration.

**Convergence condition** It is the time interval required by the learning algorithm to achieve the optimal response. In CoWSN, if the learning algorithm finds that the response does not change in the five last iterations, then the algorithm is convergent. Finally, the optimal response (the scheduling determined for $SN_{i}$) is stored.

In algorithm 2, the pseudo-code of the scheduling mechanism is presented.
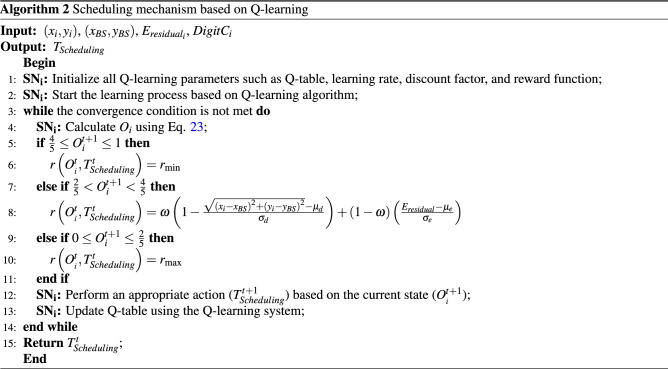


### Node replacement

After launching the network, the sensor nodes begin their activities according to the time slots specified in the scheduling round. These activities lead to energy loss of sensor nodes. In this section, we predict the death time of nodes to prevent possible holes in the network. This work does not allow that the data transmission process between nodes and the BS is disrupted. To achieve this purpose, each sensor node (for example, $SN_{i}$) updates periodically its $Priority_{i}$ based on Eq. (29) to determine its importance for replacing.

29 $$\begin{aligned} Priority_{i}=\left( 1-\frac{{{O}_{i}}}{\pi RS_{i}^{2}}\right) +\left( \frac{Packet_{size_{i}}}{Buffer_{size_{i}}}\right) \end{aligned}$$

where $\left( 1-\frac{{{O}_{i}}}{\pi RS_{i}^{2}}\right)$ is the non-overlapping area of $SN_{i}$. Note that $O_{i}$ is the overlapping area of $SN_{i}$ obtained through Eq. (13). Also, $RS_{i}$ is the sensing radius of $SN_{i}$. $\left( \frac{Packet_{size_{i}}}{Buffer_{size_{i}}}\right)$ calculates the data traffic in $SN_{i}$. $Packet_{size_{i}}$ is the number of packets in the buffer of $SN_{i}$ at a specific time. Also, $Buffer_{size_{i}}$ represents the buffer size of $SN_{i}$.

When $SN_{i}$ loses its energy so that its energy is lower than a threshold. This node sends a warning message along with its $Priority_{i}$ to the BS. Then, the BS compares $Priority_{i}$ with $P_{Threshold}$ (a threshold value for priority) to decide on the replacement of this node. Note that $P_{Threshold}$ is a constant amount so that $P_{Threshold}>0$.

- If $Priority_{i}>{P_{Threshold}}$ is larger than $P_{Threshold}$, then $SN_{i}$ has a higher importance for replacing because if $SN_{i}$ dies in the network, then the normal network operations are damaged. Therefore, the BS sends a Coverage message to the mobile node closest to $SN_{i}$ for replacing this node.
- If $Priority_{i}$ is smaller or equal to $P_{Threshold}$, then the death of $SN_{i}$ cannot disrupt the normal network operations. Therefore, the BS ignores $SN_{i}$.

Algorithm 3 presents the pseudocode of the node replacement.
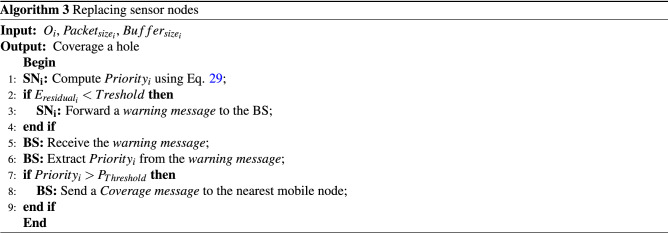


## Simulation and result evaluation

In this section, we simulate CoWSN with NS2 to evaluate its performance. The simulation results are compared with the scheme of Rahmani et al.^5^, CCM-RL^32^, and CCA^33^. We assume that the network includes 250–2000 heterogeneous sensor nodes, which are randomly distributed in the network. The nodes have different sensing ranges (i.e. 25, 30, and 35 m) and communication ranges (i.e. 50, 60, and 70 m). The network size is equal to $1000\times 1000\,\,\mathrm {m}^{2}$. When nodes are active, they consume energy equal to $57\,$mA. Also, when nodes are inactive, they consume $0.40\,\, \upmu$A. Table 3 presents simulation parameters in summary. We evaluate the performance of CoWSN in terms of four parameters, including the average number of active sensor nodes, coverage rate, energy consumption, and network lifetime.

**Table 3:** Simulation parameters.

Parameter	Value
Simulator	NS-2.35
Network size	$1000\times 1000$ m$^{2}$
Total number of nodes	250–2000
Simulation time	1200 s
Sensing radius	25, 30, and 35 m
Communication radius	50, 60, and 70 m
Initial energy of nodes	100 J
Energy consumed by active nodes	57 mA
Energy consumed by inactive nodes	0.40 $\upmu$A

### The average number of active sensor nodes

**Figure 7:**
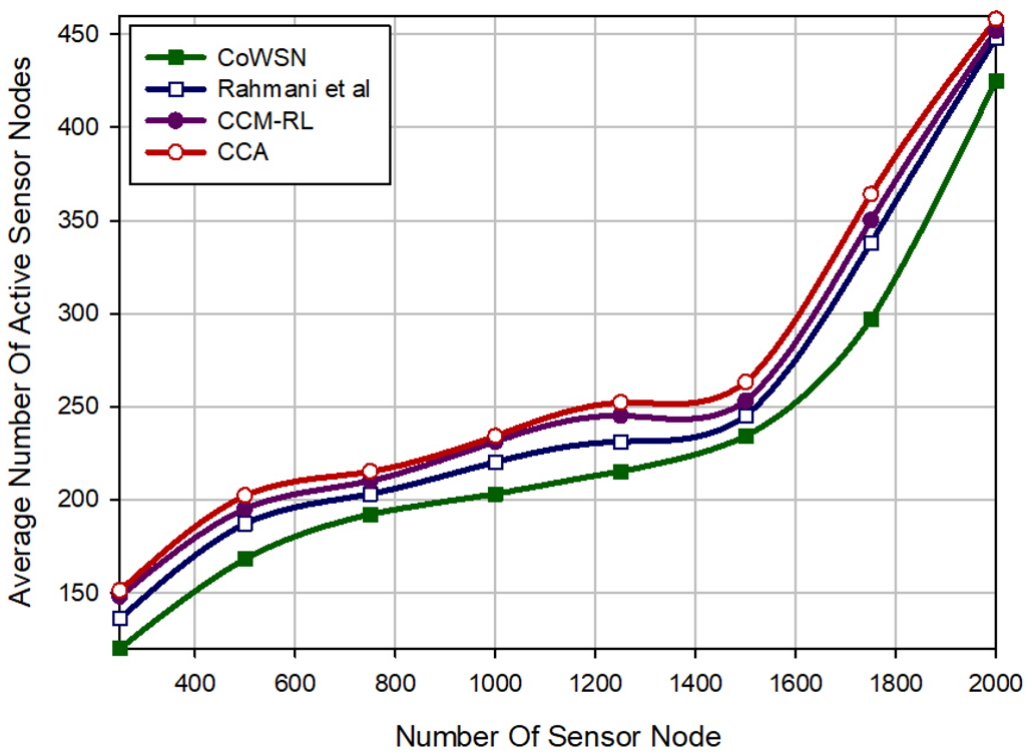
Comparison of the average number of active nodes in different schemes.

The number of active sensor nodes indicates the subsets of the active nodes selected for covering the Region of Interest (RoI). As shown in Fig. 7, CoWSN has the best performance in terms of the number of active nodes at a scheduling period. This means that CoWSN lowers the number of active nodes by 7.67%, 11.04%, and 13.32% compared to Rahmani et al., CCM-RL, and CCA, respectively. This is because CoWSN uses a Q-Learning-based scheduling mechanism to determine the activity time of the sensor nodes. CoWSN and Rahmani et al. calculate the overlap between a sensor node and its neighbors by a precise approach. Although, the scheme of Rahmani et al. has a fuzzy scheduling mechanism for calculating the activity time of nodes in the network. This method has a weaker performance than our method. Also, CCM-RL focuses only on the distance parameter when calculating the overlap of nodes. This is not a precise method and can have a lot of error. In addition, CCA does not present any approach to calculate the overlap between nodes. On the other hand, CoWSN considers two parameters, including energy and distance to the base station, to determine the best scheduling for sensor nodes. CCA focuses on the energy parameter in the scheduling process, but does not pay attention to the overlap between nodes. Furthermore, CCM-RL does not consider energy of nodes in the learning process. It is an important weakness of this method. According to Fig. 7, when the number of nodes is increasing in the network, all methods increase the average number of active nodes. Note that a coverage method cannot activate all nodes at all times because they consume high energy and die quickly, which reduces network lifetime. As a result, when the density of nodes is low in the network, the active nodes cannot cover the whole RoI. Thus, the coverage rate is reduced. But when the density of nodes is increasing, each method increases the number of active nodes. Therefore, the coverage quality of the RoI is improved.

### Coverage rate

**Figure 8:**
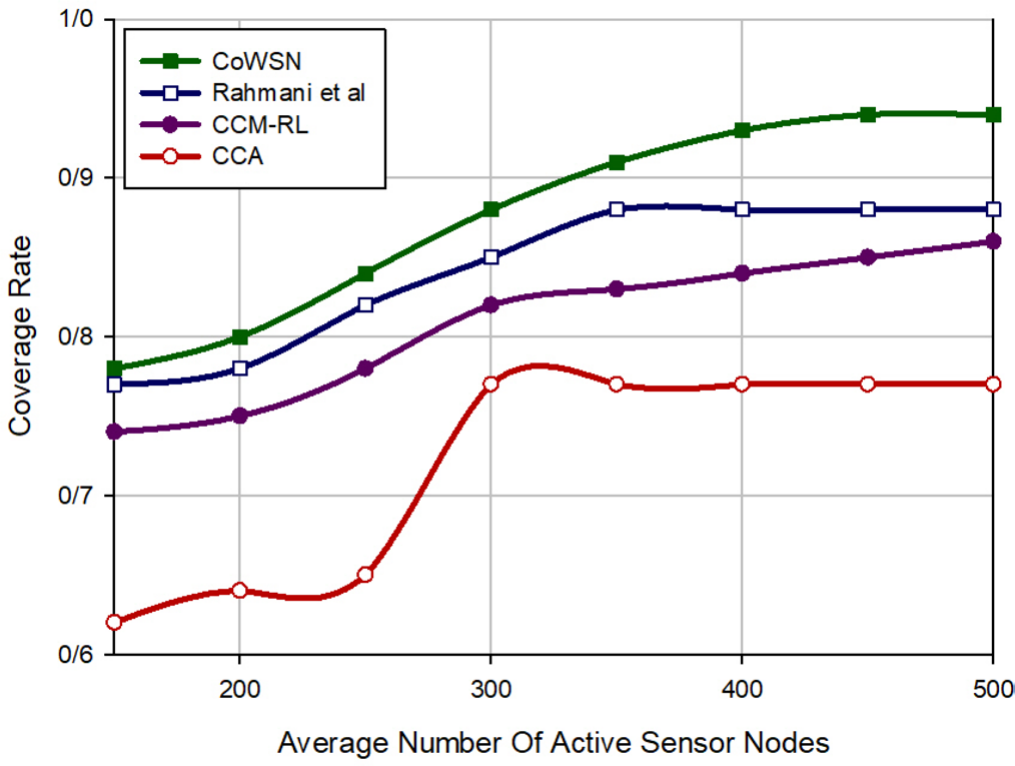
Comparison of coverage rate in different schemes.

The coverage rate is defined as the percentage of the RoI covered by active nodes. As shown in Fig. 8, CoWSN increases the coverage rate by 4.15%, 8.50%, and 21.87% in comparison with Rahmani et al., CCM-RL, and CCA, respectively. This is due to the fact that our method calculates overlapping between nodes accurately and penalizes the nodes with more overlapping. This means that they receive the lowest reward to be in the sleep mode for more slot times. This helps CoWSN to achieve the best coverage rate with the lowest active nodes in the network. Moreover, CoWSN can predict the death of sensor nodes and timely replace them to prevent coverage quality loss. Meanwhile, CCM-RL and CCA do not provide any approach to replace dead nodes. Although, the scheme of Rahmani et al. addresses this issue using SFLA. According to Fig. 8, when the number of active nodes is more than 350, CoWSN achieves a coverage rate more than 90%, which is very desirable. While the scheme of Rahmani et al. has achieved a coverage rate equal to 88% for this number of active nodes. This coverage rate is fixed and does not change when increasing the number of active nodes. In CCM-RL, the coverage rate is not constant and is improved when increasing the number of active nodes. Although, in the best mode, CCM-RL has reached a coverage rate equal to 86%. In CCA, the coverage rate is equivalent to 77% for 350 active nodes or more.

### Energy consumption

**Figure 9:**
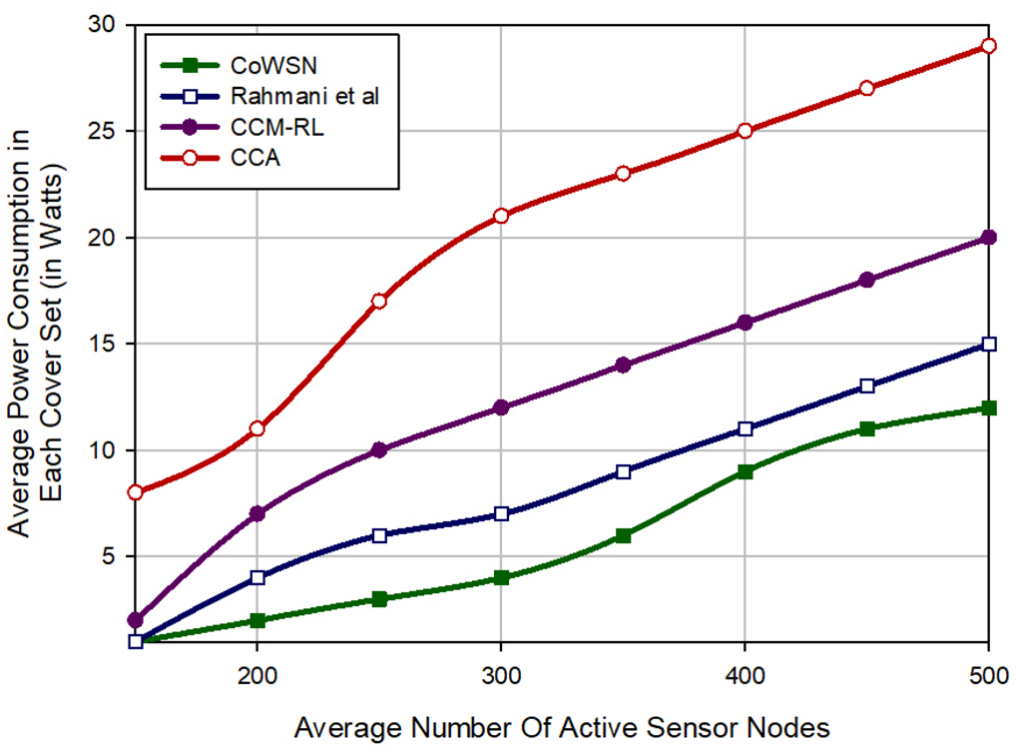
Comparison of the average energy consumption in different schemes.

As shown in Fig. 9, CoWSN reduces energy consumption by 27.27%, 51.51%, and 70.19% compared to Rahmani et al., CCM-RL, and CCA, respectively. This is because our Q-learning-based scheduling mechanism considers the energy parameter when designing the reward function. In our method, high-energy nodes receive a reward and increase their Q value to be in the active mode for more time. Moreover, low-energy nodes are penalized to do their activities in a short time. Also, the scheme of Rahmani et al. considers the energy parameter in the scheduling process of sensor nodes. However, this method has high communication overhead because it uses fuzzy logic in the scheduling mechanism. Furthermore, it uses SFLA to cover the hole created in the network. SFLA increases energy consumption in this method. CCM-RL has the third rank in terms of energy consumption compared to other methods. This scheme does not consider the energy parameter in the scheduling process. Although, it uses the sensing range customization mechanism, which helps CCM-RL to consume energy efficiently. CCA has the worst performance in terms of energy consumption because it has high communication overhead.

### Network lifetime

**Figure 10:**
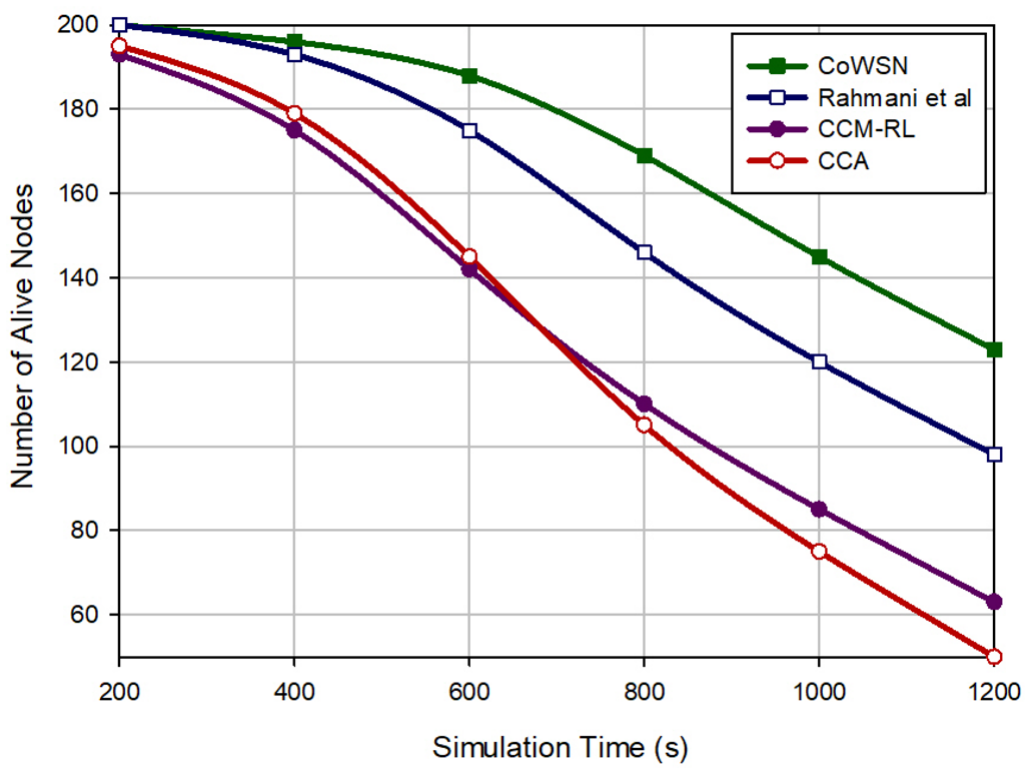
Comparison of network lifetime in different methods.

Figure 10 compares various methods in terms of network lifetime. We assume that there are 200 alive nodes in the network when doing this experiment. The nodes consume their energy over time. CoWSN improves the network lifetime by 9.55%, 32.94% and 36.32% in comparison with Rahmani et al., CCM-RL, and CCA, respectively. We described the reasons for this issue in “Energy consumption”. CoWSN tries to evenly distribute energy consumption between sensor nodes in the network because it takes into account the energy parameter in the scheduling process.

## Conclusion

In this paper, we presented an area coverage scheme called CoWSN to intelligently monitor gas and oil pipelines. The purpose of CoWSN is to reduce energy consumption, improve network lifetime, and achieve the highest coverage rate in the network. To achieve these goals, we used a digital matrix-based technique to calculate the overlap between each sensor node and its neighboring nodes. Then, we designed a Q-Learning-based scheduling mechanism to determine the activity time of each sensor node. Also, CoWSN uses a suitable strategy to timely detect the death of nodes and prevent holes in the network. To evaluate CoWSN, it is simulated with NS2 and compared with the scheme of Rahmani et al., CCM-RL, and CCA methods. Simulation results show the successful performance of CoWSN. In this paper, we have used the WSN platform (and not a real-time gas or oil pipeline network) to simulate our scheme. In the future research direction, we try to evaluate our method in real gas or oil pipeline environments and under more scenarios so that the performance of our method is further identified. Also, we seek to improve the efficiency of our method using other machine learning (ML) techniques and evolutionary algorithms (EAs) in the future.
